# Investigating Digital Patient-Reported Outcome Measures in Patient-Centered Diabetes Specialist Outpatient Care (DigiDiaS): Protocol for a Multimethod Prospective Observational Study

**DOI:** 10.2196/52766

**Published:** 2024-03-05

**Authors:** Astrid Torbjørnsen, Ingeborg Spildo, Maria Aadland Mollestad, Annesofie Lunde Jensen, Tone Singstad, Nina Mickelson Weldingh, Pål Joranger, Lis Ribu, Heidi Holmen

**Affiliations:** 1 Department of Nursing and Health Promotion Faculty of Health Sciences Oslo Metropolitan University – OsloMet Oslo Norway; 2 Department of Clinical Medicine Aarhus University Aarhus Denmark; 3 SDCA-Steno Diabetes Centre, Aarhus Aarhus University Hospital Aarhus Denmark; 4 Division of Medicine Akershus University Hospital Akershus Norway; 5 Division of Research and Innovation Department of Research Support Service Akershus University Hospital Akershus Norway; 6 The Centre for Senior Citizen Staff Oslo Metropolitan University – OsloMet Oslo Norway; 7 The Intervention Centre Oslo University Hospital Oslo Norway

**Keywords:** patient-reported outcome measures, PROMs, diabetes mellitus, DM, type 1, patient acceptance of health care, telemedicine, mobile apps, mobile phone

## Abstract

**Background:**

Living with type 1 diabetes is challenging, and to support self-management, repeated consultations in specialist outpatient care are often required. The emergence of new digital solutions has revolutionized how health care services can be patient centered, providing unprecedented opportunities for flexible, high-quality care. However, there is a lack of studies exploring how the use of digital patient-reported outcome measures (PROMs) for flexible specialist care affects diabetes self-management. To provide new knowledge on the relevance of using PROMs in standard care, we have designed a multimethod prospective study.

**Objective:**

The overall aim of this protocol is to describe our prospective multimethod observational study designed to investigate digital PROMs in a routine specialist outpatient setting for flexible patient-centered diabetes care (DigiDiaS).

**Methods:**

This protocol outlines the design of a multimethod prospective observational cohort study that includes data from electronic health records, self-reported questionnaires, clinical consultation field observations, and individual in-depth interviews with patients and diabetes health care personnel. All patients with type 1 diabetes at a designated outpatient clinic were invited to participate and use the digital PROM implemented in clinical care. Both users and nonusers of the digital PROM were eligible for the prospective study, allowing for a comparison of the two groups. Data were collected at baseline and after 12 months, including self-management as the primary outcome assessed using the Patient Activation Measure, along with the secondary outcomes of digital health literacy, quality of life, health economy, and clinical variables such as glycated hemoglobin.

**Results:**

The digital solution was implemented for routine clinical care in the department in November 2021, and data collection for the prospective study started in October 2022. As of September 6, 2023, 84.6% (186/220) of patients among those in the digital PROM and 15.5% (34/220) of patients among the nonusers have consented to participate. We expect the study to have enough participants by the autumn of 2023. With 1 year of follow-up, the results are expected by spring 2025.

**Conclusions:**

In conclusion, a multimethod prospective observational cohort study can offer valuable insights into the relevance, effectiveness, and acceptability of digital tools using PROMs in diabetes specialist care. Such knowledge is crucial for achieving broad and successful implementation and use of these tools in a large diabetes outpatient clinic.

**International Registered Report Identifier (IRRID):**

DERR1-10.2196/52766

## Introduction

### Background

Living with type 1 diabetes can impact one’s daily life [1]. Self-management is required to reduce late complications, but it can be exhausting and stressful [2], and the burden of living with type 1 diabetes often impacts a person’s mental health [3,4]. However, there is a significant variation in the needs and concerns of people with type 1 diabetes and varying perceptions of the diabetes burden. To improve patient-centered diabetes care, targeting unique needs and perspectives in both clinical care and research is necessary [5].

A recent systematic review has provided evidence for the effect of an integrated care model in diabetes care on essential patient outcomes [6], which is in line with clinical recommendations [7]. Thus, the increased involvement of patients is warranted. One way of increasing the involvement and bringing the voice of the patients forward is through “patient-reported outcomes” (PROs), that is, patients’ responses to outcomes relevant to their condition [8]. Therefore, PRO measures (PROMs) are the measures used to assess PROs systematically.

PROMs in diabetes care can improve patient-centered care by collecting information directly from patients to obtain a complete picture of the patient’s health status. Health care providers could better understand their patients’ needs and concerns and tailor care to meet these individual needs [8,9]. However, it has been challenging to select the tools for PRO measurement to ensure that they are valid and responsive to changes in patient health status [10]. In addition, the clinical value of the PROMs has not been established, and the adaptation of standardized PROMs used in research might not be straightforward because the measures are usually lengthy and time-consuming to answer for the patient and to interpret for the clinician. The use of PROMs in previous research has varied widely in terms of using one or several PROMs, using disease-specific or generic PROMs, and determining at what times or under which conditions the measures are used [11]. The use of PROMs in diabetes care has become increasingly multidimensional, focusing on a range of patient outcomes and highlighting the need for a broader multidisciplinary and shared effort in clinical practice [12].

Digital development has affected the use of digital PROMs in the last decades, offering easier and more timely access to patient reports and an easier and more timely way of evaluating patient reports for health care personnel [13,14]. Digital PROMs have been successfully implemented in various services [15], including diabetes [16,17]. Despite the many benefits of digital PROMs, the reasons for the lack of use among patients remain, including a lack of motivation, technical barriers, emotional distress, and a reduced ability to participate in a digital PROM [18].

To ensure the patients’ participation in digital PROMs as intended by the health services, research on the patients’ perspectives is essential to providing a more complete understanding of health care needs and preferences. Patients’ acceptance of digital support is crucial and can affect their engagement and adherence [19]. Furthermore, patient perception, acceptability, and engagement in designing and implementing digital health intervention evaluations remain crucial [20]. By understanding and addressing the concerns and barriers to digital solutions faced by patients who may be uncomfortable or unable to engage in digital support, we can promote more equitable access to health care services. Similarly, measuring technology acceptability after use—rather than predicted use—might provide valuable insights into users’ perceptions and experiences of technology, helping to identify areas for improvement [21].

An increasing number of patients are in need of care, with limited resources and staff to ensure their needs, which holds true in diabetes care [22]. Thus, implementing digital PROMs to support self-management in line with patient-centered care might alleviate the burden on both patients and services [23]. However, further research is needed to fully understand the interactions in these new services among the patient, the clinicians, and the digital solution to understand the effects and implications on the users.

### Aims

The overall aim of this protocol is to describe our prospective multimethod observational study designed to investigate digital PROMs in a routine specialist outpatient setting for flexible patient-centered diabetes care. Specifically, the DigiDiaS will (1) quantitatively investigate and describe the characteristics of patients with type 1 diabetes participating in a digital PROM in comparison with patients in traditional follow-up and evaluate the effect of participating in digital PROMs on clinical outcomes, self-management, diabetes distress, quality of life, and health care utilization and (2) qualitatively, through observations and qualitative interviews, assess patients’ acceptability of consultations prepared and supported by digital and flexible services using PROMs.

## Methods

### Study Design

**Figure 1:**
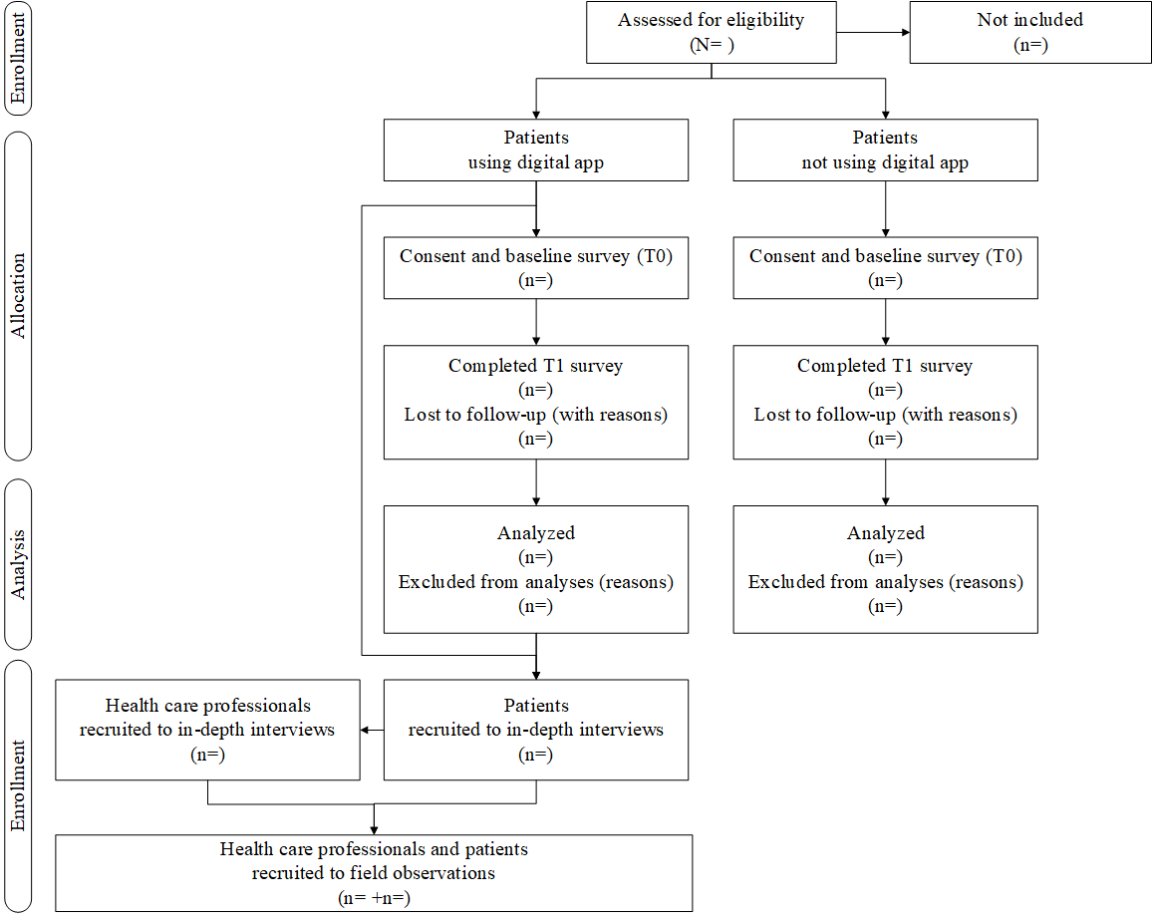
Study flow.

This protocol describes a prospective, multimethod observational cohort study to investigate the relevance and effects of digital and flexible services using PROMs. The PROM in the planned trial is based on DiabetesFlex, which was developed and implemented in Danish health care services for type 1 diabetes [16,17,24], and it has been adapted to a Norwegian digital context in preparation for this study [25]. Consenting participants will be enrolled for 12 months, with assessments at baseline (T0) and 12 months (T1), with additional data from electronic health records, field observations of clinical video, or in-person consultations, along with individual in-depth interviews with the patients and diabetes health care personnel (Figure 1). Field observations and in-depth interviews among the patients participating in the digital PROMs will be conducted at any given time for those not participating in the survey and after completing T1 for those consenting to the survey study to avoid contamination of the survey and interview data.

This study is a collaboration between Akershus University Hospital and Oslo Metropolitan University—OsloMet and will be conducted at the Endocrinological Outpatient Clinic at Akershus University Hospital.

This protocol is reported in line with the Standard Protocol Items: Recommendations for Interventional Trials (SPIRIT) guidance [26]. The reporting of the proposed study and participation in digital tools and PROMs in clinical practice will be guided by better reporting of interventions: template for intervention description and replication (TIDieR) [27] and the PRO reporting guide [28].

### Overview of the Implemented Service

#### A Digital and Flexible Service

All patients at the clinic will be offered to engage with digital and flexible services in addition to standard care. If they are interested, the digital service will be available through a mobile app on their private smartphone and will contain functions for self-monitoring, chat, video consultations, and PROMs before or between consultations. The digital PROMs implemented in this study have been described in detail elsewhere [25]. Standard care at the outpatient clinic includes a once-a-year consultation with an endocrinologist, a diabetes specialist nurse, or both. In the given situations, patients have ≥1 added consultation a year [29]. The clinic offers video-based and physical face-to-face consultations based on the patient’s preferences and needs for care through solutions approved by the hospital.

#### Digital Platform: Dignio Connected Care

Although the study does not focus on evaluating the functionality of a specific digital solution, the clinical implementation of elements from DiabetesFlex will be carried out through the digital platform Dignio Connected Care [30]. The MyDignio and DignioPrevent interfaces are presented in Multimedia Appendices 1 and 2. The patient app MyDignio allows patients to store health data and communicate with health care personnel at the clinic through self-monitoring, answering PROM questionnaires sent between consultations, communicating through a message system (chat), or engaging in video consultations with their health care personnel [31]. The health care personnel software DignioPrevent offers a flexible way of reviewing patent-reported data, using the traffic light principle to guide and prioritize which patients need attention. Dignio Connected Care allows patients to reflect on self-care and needs, take preventive actions, and prepare for consultations, both for patients to give responses of their prioritizing of needs and for the health care personnel to obtain an overview of the complexity, personalize the care, and offer the right level of expertise at the right time [25,32]. The platform facilitates a more informed dialogue between patients and health care personnel based on experience and knowledge [33]. It has also been suggested to make specialist health care more fluid and accessible to patients [34].

### Information and Training

The patients will receive information regarding the digital service in consultation, regardless of this research project. If they consent to engage in the digital service, more detailed information on the MyDignio app will be provided by the diabetes specialist nurse. An invitation to download MyDignio will be sent to the patient’s smartphone in consultation, and if they wish and have their national ID with them, they can download MyDignio immediately. The patient interface is easy and intuitive, and extensive training should not be required for most patients.

As the patient interface, the health care personnel site DignioPrevent has been developed to be simple and intuitive. The diabetes specialist nurses have been trained in using the Dignio Connected Care personnel interface DignioPrevent, by personnel from the information and communications technology unit at the university hospital, in addition to a close collaboration with personnel from Dignio Connected Care. Similarly, diabetes health care personnel will receive training in the video consultation systems used in the clinic.

### Participants

#### Patient Participants

Patients with type 1 diabetes at the Endocrinological Outpatient Clinic at Akershus University Hospital who meet the inclusion criteria are eligible for enrollment. The inclusion criteria include age ≥18 years, a type 1 diabetes diagnosis, and the ability to read Norwegian. Both users and nonusers of the MyDignio platform are eligible. The exclusion criteria are type 2 diabetes, gestational diabetes, or any cognitive impairment inflicting their ability to participate in the research project. Patients with type 1 diabetes are not eligible if they are pregnant at the time of recruitment, and they will be excluded from follow-up if they become pregnant during the 12 months because of an expected change in response caused by the pregnancy and not digital care. They will still be able to participate in the flexible digital care model.

#### Health Care Personnel Participants

In the outpatient clinic, health secretaries, diabetes specialist nurses, and physicians will be involved in the digital platform. The health secretaries send the PROM in the app to the patients along with their scheduled appointments. If the patients wish to change their scheduled appointment, the health secretaries can arrange this, but they do not handle any medical questions, assessments of PRO responses, or other messages in the system. The diabetes specialist nurses are responsible for all questions from the patients and PRO responses, including the need for a consultation, new tasks in the system, and any need for information. The physicians handle the PRO response for the yearly control in the system and otherwise consult and guide the diabetes specialist nurses if needed. Except for the yearly physician consultation, the diabetes specialist nurses are responsible for all consultations with patients with type 1 diabetes at the clinic. Thus, both diabetes specialist nurses and physicians are eligible for participation in field observations of their consultations with patients in the digital service and for in-depth interviews.

### Procedures

Eligible patients will be identified and given brief information at the outpatient clinic by health care professional staff. If the patients are interested, they will be contacted by a researcher (IS or MAM). If they consent to participate after receiving oral and written information, their written consent will be secured either through Nettskjema digital consent or through a paper-based consent form. Immediately after their consent, the patients can choose whether they will fill out the baseline questionnaire digitally through Nettskjema, paper-based in the mail at home with a free return envelope, or through a telephone interview with a researcher (IS or MAM). For consenting participants not responding to or returning the baseline questionnaire or the 1-year follow-up, an automatic email reminder will be sent, followed by a phone call reminder and a SMS text message reminder if they still do not reply. The digital consents and the digital questionnaire data will be securely stored in the Service for Sensitive Data at the University in Oslo. Paper-based consent forms and questionnaires will be securely locked in a safe for storage, and the paper-based questionnaires will be manually entered into SPSS (IBM Corp). Data from the patients’ medical records will be extracted by a researcher (IS), and 10% of the extractions will be controlled by a second researcher (MAM) to ensure valid and reliable data extraction.

Health care personnel will be recruited for 2 purposes: in-depth interviews and observations of consultations with patients engaged in the digital PROM. The personnel will be recruited from the department, and written informed consent will be secured using the same procedures as those for the patients.

### Study Outcomes

#### Overview

The primary outcome in this study is the change in self-management, as measured through the Patient Activation Measure (PAM) questionnaire after 1 year, which will then be compared between the users and the nonusers of the digital PROM. The secondary outcomes include glycated hemoglobin (HbA_1c_), quality of life, health literacy, acceptability, health economics, and the use of health service resources. Quantitative data will be collected through patients’ self-reports and the extraction of clinical variables from patients’ medical records. In addition, data on the patients’ use of MyDignio will be extracted, including how many clinical PROMs they completed during the study period. Self-reported and clinical variables will be collected among all consenting participants, regardless of their participation in the digital solution. The standardized self-reported outcome measures are presented in Table 1.

**Table 1:** Standardized self-reported outcome measures.

Domain and questionnaire and item, scale, and interpretation					Time point						
					T0		T1				
**Self-management**											
	**PAM-13^a^**										
		13 items 4-point Likert scale ranging from “strongly disagree” to “strongly agree” and “nonapplicable” Higher scores indicate higher patient activation A total of 4 activation levels in progressing difficulty: (1) belief that their role is important (0-47; items 1-2); (2) confidence and knowledge to act (47.1-55.1; items 3-8); (3) taking action (55.2-72.4; items 9-11); (4) staying on course under stress (72.5-100; items 12-13) Domains: Knowledge, beliefs, confidence, and skills related to self-managing health and improving outcomes		✓		✓					
**Diabetes distress**											
	**PAID^b^**										
		20 items 5-point Likert scale ranging from 0 (“not a problem”) to 4 (“serious problem”) The sum score multiplied by 1.25 gives a total score ranging from 0 to 100. A higher score reflects greater emotional distress. A score of ≥40 indicates severe emotional distress.		✓		✓					
**Quality of life**											
	**WHO-5^c^**										
		5 items (statements) 6-point Likert scale ranging from 0 (“at no time”) to 5 (“all of the time”) Total raw score ranging from 0 to 25 is multiplied by 4 (total score), where 0 represents worst imaginable well-being and 100 represents best imaginable well-being.		✓		✓					
	**EQ-5D-5L**										
		5 dimensions: mobility, self-care, usual activities, pain and discomfort, and anxiety and depression Each dimension has 5 levels, from “no problems” (level 1) to “extreme problems” (level 5)		✓		✓					
**Digital health literacy**											
	**HLS19-DHC-NO^d^**										
		10 items 4-point Likert scale from 1 (“very hard”) to 4 (“very easy”) with added “I don’t know” Higher scores reflect higher digital health literacy.				✓					
	**HLS-Q12^e^**										
		12 items 4-point Likert scale from 1 (“very hard”) to 4 (“very easy”) with added “I don’t know” Higher scores reflect higher digital health literacy.				✓					
**Patient acceptability**											
	**SUTAQ^f^**										
		22 items 6-point Likert scale ranging from 1 to 6, reflecting more or less agreement with the statements. High values reflect a high degree of agreement, except for 2 categories: *privacy and discomfort and care personnel concerns.*						✓			
		**Perceived benefit**									
			Enhanced care—beliefs about how health technology enhances care from health care personnel Increased accessibility—beliefs on how health technology increases access to care					✓			
		**Privacy and discomfort**									
			Concerns about the impact of the kit on the person and the safety of the information monitored					✓			
		**Care personnel concerns**									
			Beliefs about personnel skills and continuity of care					✓			
		**Kit as substitution**									
			Beliefs about health technology as an alternative to standard care					✓			
		**Satisfaction**									
			Beliefs of acceptance and satisfaction with health technology used in health care services					✓			

^a^PAM: Patient Activation Measure.

^b^PAID: Problem Areas in Diabetes.

^c^WHO-5: World Health Organization-5.

^d^HLS19-DHC-NO: Health Literacy Survey-19 Digital Health Care in Norwegian.

^e^HLS-Q12: Health Literacy Survey Questionnaire-12 item.

^f^SUTAQ: Service User Technology Acceptability Questionnaire.

#### Self-Management

To evaluate self-management, we propose the PAM short version (PAM-13) for assessing self-management through patient activation in 4 domains: knowledge, beliefs, confidence, and skills for managing one’s health [35]. The PAM-13 is suitable for the evaluation of health programs, which will enable patients to take responsibility for their own health; it contains 13 items, with scoring ranging from strongly disagree to strongly agree. The total PAM score can be divided into 4 levels: level 1 (“not believing activation is important”) and level 2 (“a lack of knowledge and confidence to take action”) indicate lower patient activations and level 3 (“beginning to take action”) and level 4 “taking action” indicate higher patient activation. The PAM-13 was developed by Hibbard et al [35,36] for working with people with and without chronic conditions, and the initial validation showed strong psychometric properties. The PAM has previously been used among people with diabetes, albeit mostly type 2 diabetes [37], and has been translated into Norwegian and validated in a previous study [38].

#### Diabetes Distress

To evaluate diabetes distress, the Problem Areas in Diabetes-20 scale is used. This is an emotional distress scale for measuring diabetes-related concepts, such as depression, social support, health beliefs, and coping style; the scale aims to identify high risk for negative effects on self-management and emotional burnout because of diabetes [39]. The Problem Areas in Diabetes-20 contains 20 items, with a 4-point Likert scale ranging from no problem to a serious problem. It has been translated into Norwegian and validated in a previous study [40].

#### Quality of Life

Quality of life will be assessed using the 5-item World Health Organization Well-Being Index, which is a measure of current mental well-being and overall quality of life over the individual’s past 2 weeks [41]. It contains 5 statements, with responses on a 6-point Likert scale that ranges from “no time” to “all of the time.” The questionnaire has been validated and applied across various study fields [41] and is available and widely used in Norwegian.

To assess quality of life with added relevance to the health economic analyses, the EQ-5D-5L, including the EQ visual analogue scale, will be used. EQ-5D includes questions about mobility, self-care, usual activities, pain, discomfort, anxiety, and depression on a 4-level scale that ranges from “no problem” to “unable to/extreme problems.” The EQ-5D visual analogue scale allows patients to rate their own overall current health. EQ-5D is a standardized generic instrument that is suitable for use in economic evaluations in health care. It was previously translated to Norwegian with population norms established [42], and it is widely applied on a global scale [43,44].

#### Digital and Health Literacy

Digital health literacy will be evaluated using the generic Health Literacy Survey-19 Digital Health Care in Norwegian (HLS19-DHC-NO). It measures the skills in using electronic tools to follow-up on one’s own health and disease, as well as the competence to use digital home-based follow-up. This scale contains 10 items that are scored on a 4-point Likert scale from very hard to very easy, in addition to an “I don’t know” category. It has recently been translated and applied to a Norwegian population survey [45] and is currently under validation.

The 12-Item Short-Form Health Literacy Survey Questionnaire is a generic measure of the ability to make informed health choices through 4 domains: access, understand, appraise, and apply health information. This scale contains 12 items, which are scored on a 4-point Likert scale that ranges from “very hard” to “very easy,” in addition to an “I don’t know” category. The 12-Item Short-Form Health Literacy Survey Questionnaire has been validated among the general Norwegian population [46].

#### Patient Acceptability of Digital Care

To assess the patients’ satisfaction and acceptability with participating in digital care, we will use the Service User Technology Acceptability Questionnaire, which has 22 items [21]. The Service User Technology Acceptability Questionnaire measures the acceptance of mobile health technology and can also be used to identify the characteristics of participants with low acceptance of technology. The responses are given on a 6-point Likert scale, ranging from strongly agree to strongly disagree. It has been translated into Norwegian and validated in a previous study [47].

#### Sociodemographic and Clinical Variables

Sociodemographic variables will be extracted from the patient records, whereas clinical variables will comprise self-reported data and data extracted from the patients’ medical records (Table 2).

**Table 2:** Sociodemographic and clinical variables.

Variables, scale, and interpretation				Time point		
				T0		T1
**Sociodemographic variables**						
	**Age**					
		Years	✓			
	**Sex**					
		Female or male	✓			
	**Education**					
		Not completed primary school (10 years) Primary upper secondary school Vocational school College or university (≤4 years) College or university (>4 years) Unknown	✓			
	**Employment status**					
		Employed 100% Student Unemployed (disability benefits or retired or other) Part-time employment (≤25%, 26%-50%, 51%-75%, 76%-99%)	✓		✓	
	**Cohabitation status**					
		Living alone yes or no	✓			
	**Ethnicity**					
		European Asian African Unknown	✓			
	**Tobacco habits**					
		Current user Previous user	✓		✓	
**Clinical variables**						
	**Diabetes duration**					
		Years	✓			
	**HbA_1c_^a^**					
		Mmol/mol	✓		✓	
	**Time in the range**					
		The last 14 days Time below range <3.9 mmol/L Time in range of 3.9-10 mmol/L Time above range >10 mmol/L	✓		✓	
	**Diabetes complications**					
		Number of diabetes late complications; Albuminuria, treated with dialysis, transplanted kidney, retinopathy, neuropathy, stroke, arterial vascular surgery, amputation, and diabetic foot ulcers	✓			
	**Equipment**					
		Pump, CGM^b^ sensor, pump and pen	✓		✓	
	**DKA^c^**					
		Never Once Several times Unknown	✓		✓	
	**Hypoglycemia in need of help**					
		Never Once Several times Unknown	✓		✓	
	**Symptomatic hypoglycemia**					
		Number of incidents, 0-90 past 30 days	✓		✓	
	**Height**					
		cm	✓			
	**Weight**					
		kg	✓		✓	
	**Lipid status**					
		LDL^d^-cholesterol, mmol/L	✓		✓	
	**Blood pressure**					
		Systolic and diastolic mm Hg	✓		✓	
**Comorbidity**						
	**CCI^e^**					
		Myocardial infarction, congestive heart failure, peripheral vascular disease, cerebrovascular accident or transient ischemic attack, dementia, chronic obstructive pulmonary disease, connective tissue disease, peptic ulcer disease, liver disease, hemiplegia, chronic kidney disease, solid tumor, leukemia, lymphoma, and AIDS	✓			

^a^HbA_1c_: glycated hemoglobin.

^b^CGM: continuous glucose monitoring system.

^c^DKA: diabetic ketoacidosis.

^d^LDL: low-density lipoprotein.

^e^CCI: Charlson Comorbidity Index.

#### Utilization of Health Care Resources

Data regarding the utilization of health care resources will be collected, including the type of health care services provided, frequency of utilization, participation, and any engagement in digital services. The variables are presented in Table 3.

**Table 3:** Utilization of digital solutions and health care resources.

Variables, scale, and interpretation				Time point		
				T0		T1
**Health care use**						
	**Consultation type**					
		Physical, video, or telephone			✓	
	**Attendance**					
		Number of on-attendance and number of late cancelations			✓	
	**Health care profession**					
		Number of consultations with a physician, a nurse, and a nutritionist			✓	
	**GP^a^ visits**					
		Number of GP consultations			✓	
	**Absence from work**					
		Days of absence			✓	
**Patient involvement**						
	**Patient involvement and shared decision-making**					
		6 items reflecting decision-making, communication, and interaction 5-point Likert scale from 1, “absolutely not” to 5, “to a very high degree,” with an added “I don’t know” Higher scores reflect a higher feeling of involvement	✓		✓	
**Digital user data**						
	**Participation in the digital outpatient care**					
		Yes or no, including start date	✓		✓	
	**PROMs^b^ completed**					
		Type of measures and frequency, number			✓	
	**Messaging services**					
		Frequency of use and number			✓	
	**Use of other mobile health apps**					
		2 items reflecting use and frequency Including open-ended responses	✓		✓	

^a^GP: general practitioner.

^b^PROM: patient-reported outcome measure.

The items reflecting patient involvement [48] and patient role [49] have been previously adapted from their original form and used in the DiabetesFlex study [16,17]. For this project, the items adapted in the DiabetesFlex study have been translated from Danish by the project team into Norwegian and then back-translated by a bilingual Danish researcher not affiliated with the project. The translation was then reviewed by ALJ, AT, and IS, and small adjustments were made before a translation consensus was reached.

#### Health Economy

To estimate the cost-effectiveness of the digital care model, we will conduct a cost-utility analysis based on decision-analytic modeling [50,51]. Those using the digital PROMs will be compared with those continuing with the standard follow-up. Health benefits will be measured using the EQ-5D-5L at baseline and at 12 months. Data for health care resource use will be collected using survey data on general practitioner visits, visits to nurses and other health care professionals, and absences from planned consultations and hospital admissions (Table 3). The time used for training will be based on experiences from implementing the digital care model. Microsimulations will be used to estimate the cost of traveling and other patient expenses. The unit costs will be based on the Norwegian reimbursement systems, the marked prices, and the literature. The results will be reported as incremental cost-effectiveness ratios with credibility intervals and cost-effectiveness acceptability curves. We use probabilistic sensitivity analysis to estimate the uncertainty caused by parameter uncertainty and use deterministic sensitivity analysis to assess the effect of changes in resource use, unit cost, and assumptions [52].

#### Qualitative Outcomes

The qualitative outcomes of this proposed project include in-depth interviews with patients with diabetes and diabetes health care personnel and observations of consultations between diabetes health care personnel and patients participating in the digital care model. The semistructured in-depth interviews will aim to explore patients’ acceptability of digital communication and how patients and diabetes health care personnel utilize the technology. The same patients will be observed and interviewed after obtaining their written informed consent. The interview guide for patients and health care professionals can be found in Multimedia Appendices 3 and 4, respectively.

### Sample Size

The proposed study will evaluate the relevance and effects of digital services in practice without participants being randomly drawn to a control group and, therefore, excluded from the possibility of using technological solutions in clinical follow-up. Therefore, we can assume that there is a difference between patients who choose to engage in digital care and those who choose traditional health care services. It is impossible to foresee or decide how many patients will participate through the digital PROM, but we expect more patients in the group of users of the digital service. To statistically identify the similarities and differences between the groups, we consider a 10% alteration in PAM-13 to be clinically significant, such as a change of ≥4 points. To identify a clinically relevant change to this extent, a minimum of 32 participants is required in each group. To account for potential dropouts, we will continue the recruitment of participants until we have included the required number of at least 35 participants in the control group. Consenting patients will be compared based on their chosen group, that is, engaged or not engaged in digital care. If they choose to continue with standard care at the clinic, they will contribute data for comparison purposes after providing their consent. Recruitment will continue for an estimated year, or until nearly all patients in the clinic have been offered the digital solution.

We will use purposive sampling for the qualitative interviews and the observations [53] when recruiting patients and health care personnel for the qualitative study. Approximately 25 patients participating in the digital care model are regarded as sufficient. Patient participants will be included based on purposeful sampling to gain variations in age, gender, and HbA_1c_ levels. Thus, the number of patients included will depend on the findings of a constant comparative analysis. The sample size for diabetes health care personnel depends on the consenting number of health care personnel from the outpatient clinic under study.

### Analysis

#### Statistical Analysis

The baseline (T0) and follow-up (T1) variables will be descriptively presented, whereas continuous variables will be analyzed using the median and range if the data are skewed and the mean and SD for normally distributed data. Categorical data will be presented as counts and percentages. The mean change will be estimated by subtracting the baseline scores from the follow-up scores. Any differences in mean changes in short- and long-term variables will be modeled using an ANOVA. To adjust for possible confounders, logistic regression models will include age, gender, and education. We will assess the number of patients tested for eligibility, declined to participate, lost to follow-up, and included in the analysis.

#### Qualitative Analysis

In the qualitative analysis, we will use interpretive description as a methodology [53-55]. Interpretive description aims to generate a practical understanding of the importance of applied disciplines within their context [53]. The interpretive description analysis will be performed in parallel with the data collection. All data from the transcribed interviews and field notes from the participant observation will be included in the analysis. Interpretive description analysis is an inductive, open, and exploratory approach that includes a constant comparative analysis. Hence, it builds from specific data toward a broader generalization. This analysis will lead to the identification of final themes describing patients’ acceptability of digital communication and how patients and diabetes health care personnel use the technology [53]. The NVivo software is used for data management, coding, and analysis [56]. The initial coding phase will be broad-based inductive coding into categories, followed by fine-tuned coding and interpretation of the data.

### User Involvement

In this study, health care personnel will participate alongside patients and stakeholders to adapt the DiabetesFlex to a Norwegian context, resulting in a set of PROM items that may be highly relevant to the patients. On the basis of their answers, health care personnel can triage their patients to offer suitable treatment when needed through a traffic light model. User involvement regarding the project’s development has been described elsewhere [25]. In addition, a reference group for the project will be established, containing individuals with type 1 diabetes, diabetes specialist nurses, endocrinologists, management, and researchers.

### Ethical Considerations

The Data Protection Office approved the study at Akershus University Hospital (2022_125). Patients at the outpatient clinic will be screened for eligibility for the study by health professionals, and all patients meeting the inclusion criteria will be offered the digital PROM. Therefore, a randomized controlled trial is impossible, and the proposed study has no control group. However, those declining to engage in digital tools will act as a comparative group in the analysis. All participants will provide their written informed consent before the study starts. All data will be securely stored in Services for Sensitive Data. Data shared in the MyDignio app will be encrypted and stored according to legislation on the privacy and secure storage of sensitive health information. The Data Protection Office performed a comprehensive risk assessment analysis of the technology upon initiation, and the research team completed a risk assessment analysis of the risks related to the conduct of the research before the start of the study. SIKT—the Norwegian Agency for Shared Services in Education and Research—will be notified per the Norwegian protocol to assess project data protection and information security.

To ensure data safety, patients must use their national ID to identify themselves, either through BankID or MinID, both to provide their digital consent and self-report on the digital questionnaires of the research evaluation and to log in when using MyDignio.

## Results

The study received funding in March and October 2022 from Oslo Metropolitan University–OsloMet internal funding. The digital solution was implemented in clinical care at the department in November 2021, and the first participant was enrolled in the research project with a completed T0 on October 27, 2022. As of September 6, 2023, a total of 220 patients have been enrolled in the project, of which 84.5% (n=186) are digital users and 15.5% (n=34) are nonusers in the comparative group. The data collection is projected to end during 2024.

## Discussion

### Overview

We anticipate that this study will generate knowledge on the relevance and effects of participation in digital PROMs for communication and self-management and about the characteristics of users compared with nonusers of the digital PROM. Evaluating the use of digital follow-up might facilitate the need for further development of the tools based on actual clinical use. Finally, investigating the communication between patients and their health care personnel will increase the understanding of how technology impacts consultation.

### Significance of the Study

Although diabetes care has faced considerable improvements in medical equipment facilitation and glycemic control, there is a need for more patient-centered and flexible care using recent developments in digital PROMs in routine care [11,12]. To do so, the possibilities of flexible digital care using PROMs in clinical care must be investigated. The study described in this protocol aims to provide knowledge regarding the characteristics of patients with type 1 diabetes engaged in a digital PROM, their effect on participating in digital PROMs, the patients’ acceptability of consultations prepared and supported by digital and flexible services using PROs, and how these services affect health care personnel.

Although previous research from Norway provides valuable insights into the use of PROMs in clinical diabetes consultations through the DiaPROM study, their findings highlight the need for further investigation into implementation challenges and patient acceptability [57]. In the Danish DiabetesFlex study, PROMs in the diabetes outpatient clinic had a positive impact on patients’ management of their diabetes and their responsibility for care plans. Compared with standard care, using PROM in flexible visits improved diabetes-related well-being and decreased face-to-face visits while maintaining safe diabetes management [16,24]. Using a PROM, patients were encouraged to reflect on their diabetes management; this led to a more tailored and individualized treatment approach and made the consultations more flexible, allowing for a broader dialogue between patients and health care providers [24]. Similarly, diabetes care support is crucial when living with diabetes [58]. Using PROMs in diabetes specialist care can improve communication, enhance patient engagement in their care, and improve patient outcomes. Nonetheless, it remains necessary to explore the acceptability of the use of digital PROMs to prevent inequality in health for patients who do not engage in digital health care and, as such, do not receive the intended care [18], while also identifying the barriers preventing these patients from accessing care and exploring alternative ways of delivering health care services and support. This study will investigate the effects and acceptability of a digital PROM, emphasize the potential benefits and barriers by further exploring the impact of using a PROM in a diabetes outpatient clinic, and, as such, add knowledge to existing evidence.

### Limitations

In evaluating the implementation of digital tools and PROMs, a randomized controlled trial could be considered the gold standard to minimize confounding variables and provide stronger evidence. In this case, to design a control group, we would have to withhold the tools from patients in the clinic, which could be considered unfair clinical practice in the clinic. We could have chosen to have a control group at another hospital. However, practices in endocrinology outpatient clinics are changing, making it difficult to find a hospital department administering the usual care. Therefore, this study has been planned as an observational quasi-experimental design, with its limitations being potential confounding variables and bias. This is a novel study, and we plan to include numerous participants with a long follow-up period. We will minimize bias and confounding by including patients engaged in digital PROMs and patients choosing traditional care, controlling for known confounders, and using appropriate statistical methods to analyze data in collaboration with a statistician.

A potential limitation, according to the qualitative part of the study, is related to the conduct of the observations of consultations with patients and being interviewed about their practice, with a potential risk for reactivity of social desirability bias. Diabetes health care personnel might modify their behavior while being observed to conform to expectations about performance. Similarly, they could provide responses in the interviews that they believe are socially acceptable, rather than giving honest and accurate answers. It will be important for the researchers to establish trust and ensure that the responses are kept confidential and not used to evaluate performance.

### Conclusions

There is a need for elaborate knowledge on patient participation in digital tools using PROMs in diabetes specialist care. A multimethod prospective observational cohort study can provide valuable insights into the effectiveness and acceptability of PROM digital tools, aiming for a broad measurement of their full-scale implementation in a large diabetes outpatient clinic.

## Appendix

Multimedia Appendix 1 Screenshot of the MyDignio interface.

Multimedia Appendix 2 Screenshot of the DignioPrevent interface.

Multimedia Appendix 3 Interview guide—patients.

Multimedia Appendix 4 Interview guide—health care professionals.

## Abbreviations

HbA_1c_: glycated hemoglobin
HLS19-DHC-NO: Health Literacy Survey-19 Digital Health Care in Norwegian
PAM: Patient Activation Measure
PRO: patient-reported outcome
PROM: patient-reported outcome measure
SPIRIT: Standard Protocol Items: Recommendations for Interventional Trials
