# Supplementary material for: Investigating Digital Patient-Reported Outcome Measures in Patient-Centered Diabetes Specialist Outpatient Care (DigiDiaS): Protocol for a Multimethod Prospective Observational Study
Source: JMIR Res Protoc. 2024 Mar 5;13:e52766. doi: 10.2196/52766 (PMC10951827; doi:10.2196/52766)
Supplement: Multimedia Appendix 1 [file resprot_v13i1e52766_app1.docx]

  
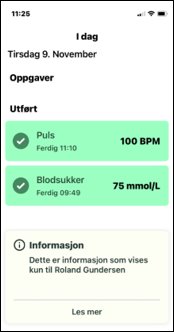


Appendix Figure 1a. Screenshot of MyDignio task view.


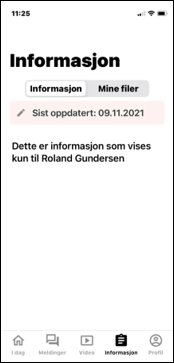


Appendix Figure 1b. Screenshot of MyDignio information view.


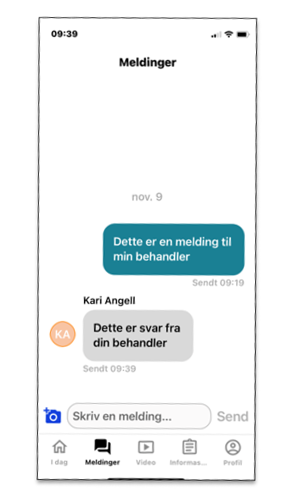


Appendix Figure 1c. Screenshot of MyDignio chat
