# Supplementary figures and images for: Investigating Digital Patient-Reported Outcome Measures in Patient-Centered Diabetes Specialist Outpatient Care (DigiDiaS): Protocol for a Multimethod Prospective Observational Study
Source: JMIR Res Protoc. 2024 Mar 5;13:e52766. doi: 10.2196/52766 (PMC10951827; doi:10.2196/52766)

 
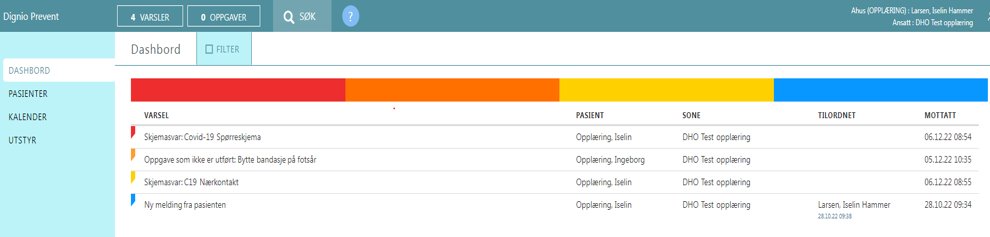


Appendix Figure 2. DignioPrevent Dashboard

Supplement: Multimedia Appendix 2 [file resprot_v13i1e52766_app2.docx]
