# Supplementary material for: Investigating Digital Patient-Reported Outcome Measures in Patient-Centered Diabetes Specialist Outpatient Care (DigiDiaS): Protocol for a Multimethod Prospective Observational Study
Source: JMIR Res Protoc. 2024 Mar 5;13:e52766. doi: 10.2196/52766 (PMC10951827; doi:10.2196/52766)
Supplement: Multimedia Appendix 3 [file resprot_v13i1e52766_app3.docx]

**Appendix 3. Interview guide – Patients**

| **Theme** | **Introduction** | **Questions** |
| --- | --- | --- |
| Introduction | Thank you for participating in this interview!   - Purpose - Data processing and recording - Content - Estimated time frame | - Do you have any questions before we start? |
| The patient  (Resources, coping and self-management) | We would like to know more about what living with diabetes is like for you | - Can you tell us about a normal day and the things you do to manage your diabetes? |
| Utilising health technology  (Digital competence) | We would like to gain a deeper understanding of how you incorporate technology in the management of your diabetes | - Can you recount your most recent experience using health technology as part of your (self)treatment? - What are your thoughts on utilising digital solutions to manage your own health? |
| Use of the MyDignio app  (Acceptability, satisfaction, usability) | We would like to hear a bit about your use of the MyDignio app | - When and how were you introduced to the app? - What guidance did you receive on using the app? - Can you describe how you have used the app? How has it been? - Could you provide an example of a time when you communicated through the app? How did you find this experience? |
| PROM  (Acceptability, satisfaction with PROM-forms) | Before consultations, you receive a form to fill out in MyDignio. We are interested in learning about your experiences with the utilisation of these forms. | - What information have you received about the forms before consultations? - Can you describe what it was like to fill out the forms? Where were you, and what were you doing? - What value does filling these forms have for you? - How have the forms been discussed and used during consultations? - How do the forms impact your interaction with the nurse/doctor? |
| Video consultations  (Communication, satisfaction) | We would like to know a bit about your experience with video consultations (if relevant) | - Can you tell us about the last time you had a video consultation? - How do you feel about having consultations over video? - How would you describe your experience when interacting with healthcare professionals through video? - When do you choose video consultations over in-person appointments? - Where have you conducted the consultation(s)? |
| Utilisation of the technology | We would like to learn more about your personal experience with utilising the technology and your thoughts on potential enhancements to these solutions | - Have the digital services of the outpatient clinic affected your daily life? Can you provide an example? - Do you find the digital solutions useful? If so, in what specific ways do you see them benefiting you? - Are there any aspects of the digital solutions that you feel are lacking? - Do you consider any features or elements of the solutions to be unnecessary? - How has your experience been with the technology itself? - Can you tell us a bit about your follow-up at the outpatient clinic before and after the digital solutions were implemented (if relevant)? - What would the perfect follow-up from the outpatient clinic look like to you? |
| Summary | To wrap up, I have a few summarising questions.  Thank you so much for being able to participate! | - Is there any information or topics we haven't covered today that you believe I should be aware of? - Is there anything you would like to say or add? - Can I contact you again for another interview if needed? |
