# Supplementary material for: Investigating Digital Patient-Reported Outcome Measures in Patient-Centered Diabetes Specialist Outpatient Care (DigiDiaS): Protocol for a Multimethod Prospective Observational Study
Source: JMIR Res Protoc. 2024 Mar 5;13:e52766. doi: 10.2196/52766 (PMC10951827; doi:10.2196/52766)
Supplement: Multimedia Appendix 4 [file resprot_v13i1e52766_app4.docx]

**Appendix 4. Interview guide – Healthcare professionals**

| Theme | Introduction | Questions |
| --- | --- | --- |
| Introduction | Thank you for participating in this interview!   - Purpose - Data processing and recording - Content - Estimated time frame | - Do you have any questions before we start? |
| The healthcare professional | First, we want to learn more about you and your role at the outpatient clinic | - Can you tell us a bit about that? |
| Utilising health technology | We would like to hear about your overall experiences and thoughts regarding the utilisation of health technology  (Glooko or other solutions) | - Could you elaborate on how, and in specific situations, you integrate health technology into your work? - What are your overall sentiments regarding the utilisation of digital tools to address health challenges? |
| Implementation of digital solutions | Over the past few years, the outpatient clinic has implemented Dignio connected care and video consultations for patient follow-up. We are interested in learning about your experience with the implementation of these solutions | - Can you describe how the solutions have been implemented in the clinic? - What has the training provided for using these digital solutions been like? - Do you feel comfortable and confident when using these digital tools in your everyday work? Why (not)? - Can you describe how other digital solutions have been implemented in the outpatient clinic in the past? |
| Dignio Prevent | We would like to learn more about your experiences with using Dignio when communicating with patients | - Can you describe the last time you used Dignio Prevent? What was it like to use the solution? - How do you introduce the app to new patients? - How do you think communicating through Dignio works? Can you give an example? |
| PROM | Patients enrolled in MyDignio will receive a form prior to their consultation. We are interested in learning about your experiences with the utilisation of these forms | - Do you provide information about the forms patients receive before a consultation? If so, what information? - How do you prepare when there is a completed form? - Could you provide an example illustrating the differences in the content of a consultation when the form is completed in advance compared to a consultation where the form is not filled out beforehand? - How do the forms affect the interaction between you and the patient? - Have you gathered any insights regarding how patients perceive the process of completing these forms? |
| Video consultations | We would like to know more about your experience of using video consultations | - Can you describe the last time you had a video consultation? - How would you assess the quality of healthcare you deliver through video consultations compared to in-person visits? - How do you feel about communicating with patients over video? |
| Utilisation of the technology | We would like to learn more about your personal experience with utilising the technology and your thoughts on potential enhancements to these solutions | - Can you remember the last time these digital solutions affected your everyday work? Can you tell us more about that? - Do you find the digital solutions useful? If so, in what specific ways do you see them benefiting you? - Are there any aspects of the digital solutions that you feel are lacking? - Do you consider any features or elements of the solutions to be unnecessary? - How has your experience been with the technology itself? - If you had the opportunity to design the follow-up tools according to your ideal specifications, what would they be like? |
| Summary | To wrap up, I have a few summarising questions.  Thank you so much for being able to participate! | - Is there any information or topics we haven't covered today that you believe I should be aware of? - Can I contact you again for another interview if needed? |
